# Lymphocyte to C-reactive protein ratio could better predict the prognosis of patients with stage IV cancer

**DOI:** 10.1186/s12885-022-10145-x

**Published:** 2022-10-20

**Authors:** He-Yang Zhang, Hai-Lun Xie, Guo-Tian Ruan, Qi Zhang, Yi-Zhong Ge, Xiao-Yue Liu, Meng Tang, Meng-Meng Song, Shi-Qi Lin, Ming Yang, Xiao-Wei Zhang, Hong-Xia Xu, Chun-Hua Song, Han-Ping Shi

**Affiliations:** 1 Department of Gastrointestinal Surgery/Department of Clinical Nutrition, Beijing Shijitan Hospital, Capital Medical University, 10 Tie Yi Road, Beijing, 100038 China; 2 Beijing International Science and Technology Cooperation Base for Cancer Metabolism and Nutrition, Beijing, 100038 China; 3 Key Laboratory of Cancer FSMP for State Market Regulation, Beijing, 100038 China

**Keywords:** Systemic inflammation, Lymphocyte-C-reactive protein ratio, Cancer, Prognosis, Patients with stage IV cancer

## Abstract

**Background:**

Systemic inflammation is currently regarded as a hallmark of cancer. This study aimed to accurately clarify the prognostic value of various inflammatory markers in patients with stage IV cancer.

**Methods:**

This study assessed 2,424 patients with cancer diagnosed with cancer in tumor, node, metastasis (TNM) stage IV. After evaluating the predictive value of 13 inflammatory indicators for patient prognosis using the C index, the lymphocyte C-reactive protein ratio (LCR) was selected to elucidate the prognostic and predictive values in patients with stage IV cancer. Kaplan–Meier and Cox proportional hazards regression models were used to analyze long-term survival.

**Results:**

A total of 1,457 men (60.1%) and 967 women (39.9%) diagnosed with TNM stage IV cancer were enrolled. A ratio of 2,814 was defined as the optimal cut-off value for the LCR. The LCR was the most accurate prognosis predictor for patients with stage IV cancer among the 13 inflammatory nutritional markers evaluated. The multivariate-adjusted restricted cubic spline plot suggested that LCR had an L-shaped dose–response association with all-cause mortality risk. Patients with lower LCR levels tended to present with worse prognoses. Kaplan–Meier curves and log-rank test results showed that the high LCR groups (LCR ≥ 2,814) exhibited a better prognosis, whereas patients with stage IV cancer of different sex and tumor types (for example, gastrointestinal tumor, non-gastrointestinal tumor, and lung cancer) had a worse survival time.

**Conclusion:**

The LCR score can be regarded as a stable and useful biomarker to predict prognosis in patients with TNM stage IV compared to other evaluated inflammation indicators.

**Supplementary Information:**

The online version contains supplementary material available at 10.1186/s12885-022-10145-x.

## Background

According to GLOBOCAN 2020 estimates, there were 19,292,789 new cancer cases worldwide, and 9,958,133 patients with cancer died in 2020. Lung cancer remains the leading cause of cancer-related deaths, with an estimated 1,796,144 deaths, accounting for 18.0 percent of total cancer deaths, followed by colorectal cancer (9.4%), liver cancer (8.3%), gastric cancer (7.7%), and breast cancer (6.9%) [1]. Cancer is the second leading cause of death in the world [1]. Therefore, there is an urgent need to explore the pathogenesis, early prevention, and diagnosis of cancer.

Despite advances in tumor screening, detection methods, and surgical modalities, the ability to predict long-term prognosis in patients with stage IV cancer after tumor resection remains poor. A growing number of studies have shown that systemic inflammatory response is correlated with cancer survival and may serve as a prognostic marker for human malignancies [2, 3]. As the seventh hallmark of cancer, systemic inflammation has been confirmed to be closely related to the development and metastasis of many malignant tumors [4, 5]. There are usually inflammatory factors present in the tumor microenvironment, and inflammation provides possible conditions for tumor occurrence [6]. However, it is unclear which components of the systemic inflammatory response can best predict survival rates in patients with stage IV cancer.

To further evaluate inflammation and its prognosis, a prognostic score based on inflammation was developed, including the neutrophil-to-lymphocyte ratio (NLR) [7], the platelet to lymphocyte ratio (PLR) [8], the Prognostic Index (PI) [9], the Prognostic Nutritional Index (PNI) [10], the Glasgow Prognostic Score (GPS) [2] and the modified Glasgow prognostic score (mGPS) [11]. Recently, another measurement method of the systemic inflammatory response, the lymphocyte to C-reactive protein (CRP) ratio (LCR) has been proposed for patients with rectal cancer, colorectal cancer, and gastric cancer [12–14]. As a promising marker, LCR has demonstrated higher ability in predicting cancer surgery and oncological outcomes in gastric cancer, rectal cancer, and cholangiocarcinoma compared to NLR, CRP, nutritional risk index (NRI), PLR, PI, PNI, GPS, and mGPS [12–17]. However, the prognostic potential of LCR in patients with advanced cancer has not been explored. In this study, we aimed to explore the prognostic value of LCR in patients with stage IV cancer and compare its prognostic predictive power with existing prognostic markers in patients with stage IV cancer.

## Methods

### Study population and design

**Fig. 1:**
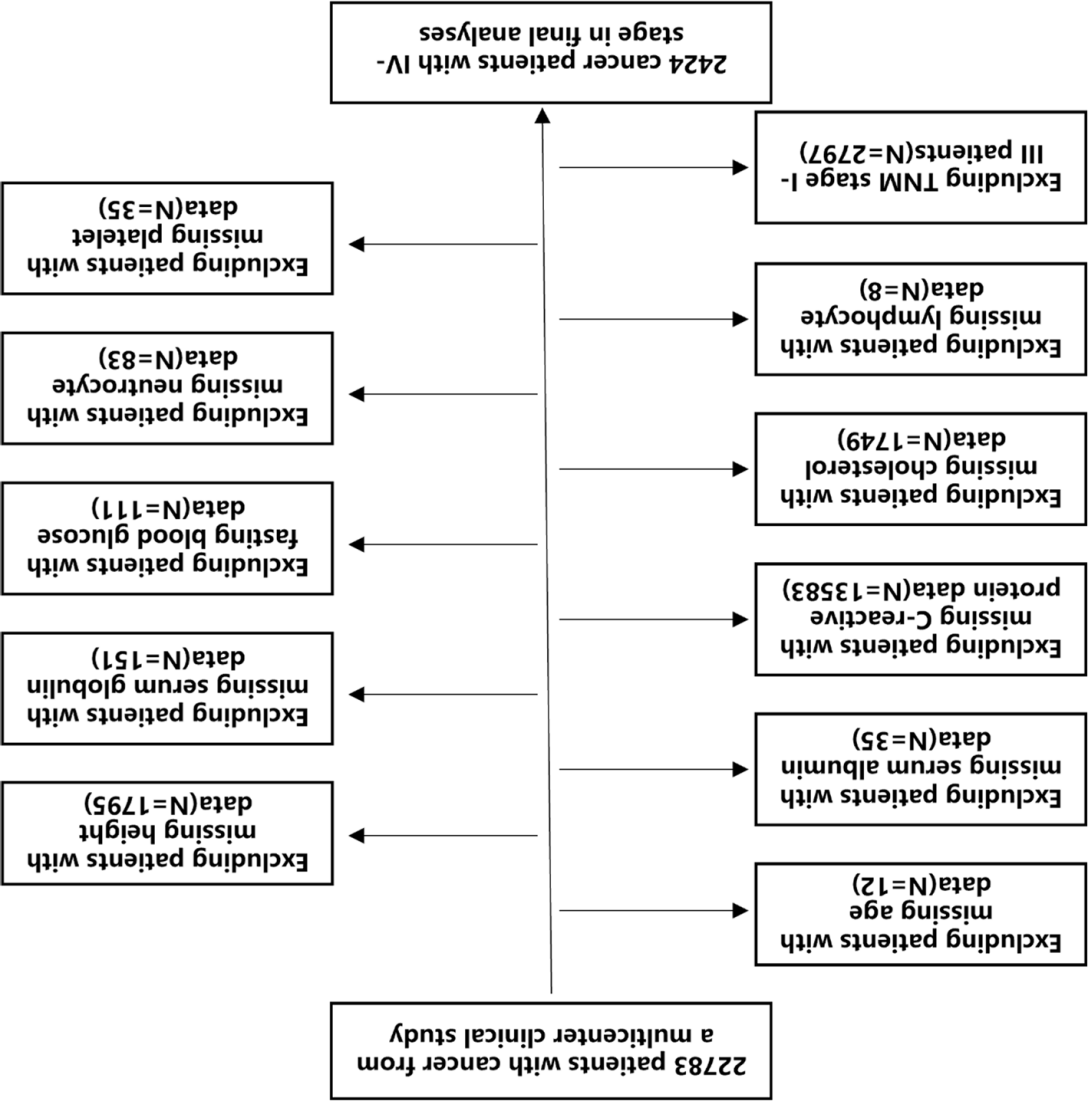
Flow chart of the study design

This multicenter observational study was based on the Common Cancer in China (INSCOC) cohort (registration number: ChiCTR1800020329; http://www.chictr.org.cn), a prospective cohort that collected data from multiple centers in China; study design, The methodology and research development process have been described previously [18]. The cohort included 2424 patients aged 18–95 years with pathologically diagnosed solid malignancies, a total of 1457 men (60.1%) and 967 women (39.9%), with a mean age of 60.18 years. Patients with multiple hospitalizations for cancer treatments (all therapies, including surgery, chemotherapy, radiotherapy, and others) were treated as one case; the baseline data from the first assessment were analyzed. Patients with serious active infections, continuous anti-inflammatory use in the past 6 months, or acquired immunodeficiency syndrome were excluded. In addition, we excluded patients with cancer TNM stages I, II, and III. Figure 1 shows a flowchart of the study screening process. The study was approved by the institutional review boards of Shijitan Hospital, and written informed consent was obtained from all the participants. All methods were carried out in accordance with relevant guidelines and regulations.

### Patient characteristics

Patient age, sex, body mass index (BMI), primary tumor type, TNM stage, smoking history, drinking history, and family history were collected from electronic medical records. The patients-generated subjective nutritional assessment (PG-SGA) and karnofsky Performance Status Scale (KPS) were evaluated and recorded by trained staff at baseline. Blood samples were collected within 24 h of admission after fasting overnight. The following data were collected from blood analysis: hemoglobin (Hb), serum albumin, PLT, and CRP levels, and other serological indicators. To eliminate differences caused by different laboratory equipment, all measurements were standardized.

### Outcomes and follow-up

All patients were followed-up regularly via telephone or outpatient visits, and clinical result information was collected. Telephone follow-up mainly included inquiries about survival and treatment, and outpatient follow-up mainly included physical examination, blood drawing, imaging, and an endoscopic examination when necessary.

The primary endpoint of this study was overall survival (OS), which was defined as the time interval between the first clinical evaluation and death, withdrawal from the study, final follow-up, or last contact. Secondary endpoints included length of stay (LOS), cost, and the KPS score (a self-scoring of health status with a total score of 100 points and 10 points per level). The 90-day outcomes were defined as all deaths within 90 days of patient enrollment in this study.

### Statistical analyses

The demographic characteristics of the study population were calculated, with continuous variables expressed as either mean ± standard deviation or median and interquartile range. Categorical variables are presented as numbers and percentages (n, %). Comparisons of differences between groups were conducted using the independent Student’s t-test or non-parametric tests for comparing continuous variables, and Chi-square test or Fisher’s exact test for comparing categorical variables. The Harrell C-index and area under the time-dependent curve (AUC) were calculated using continuous variables to evaluate and compare the predictive ability of inflammatory nutritional and anthropometric indicators for patient survival. Covariates and potential confounders were selected based on the results of previous studies. Restricted cubic spine (RCS) function with 3 knots was performed to evaluate the effects of continuous LCR on survival. Univariate and multivariate Cox regression analyses were used to evaluate the hazard ratios (HRs) and 95% confidence intervals (CIs) for important prognostic factors based on OS. A sensitivity analysis, excluding patients who died within 6 months of enrollment, was performed. Kaplan–Meier curves and log-rank tests were used to evaluate time-patient survival trends and compare survival between groups. Logistic regression models adjusted for different variables were used to assess the association of markers with patients’ daily functions and short-term outcomes. Differences were considered statistically significant for two-sided *p*-values < 0.05. Reported *P* values were not corrected for multiple testing. All statistical analyses were performed using the R software (version 4.1.1; The R Project for Statistical Computing, Vienna, Austria).

## Results

### Study participants and characteristics

We enrolled 2,424 (1,457 men, 60.1%; 967 women, 39.9%; mean age 60.18 years) patients with cancer diagnosed with TNM stage IV (Fig. 1). Optimal cut-off point values for all inflammatory nutritional indicators were obtained according to OS and using maximum selection rank statistics. The optimal critical value for LCR was determined as 2,814 (See Additional file 1).

Next, we assessed the relationship between clinicopathological factors and LCR in patients with stage IV cancer. Patients in the low LCR group (≤ 2,500) were more likely to be male (*p* < 0.001), older (*p* < 0.001), and to have a low BMI (*p* = 0.002) and various cancers such as lung cancer (*p* < 0.001), gastric cancer (*p* < 0.001), and liver cancer (*p* < 0.001) than those in the high LCR group (Table 1). In addition, these patients were more likely to have low PG-SGA scores, high KPS scores, high albumin Hb levels, and RBC levels, and low PLT levels (Table 1).

**Table 1:** Baseline characteristics of the study population

Characteristic	Overall	High LCR	Low LCR	*p*-value
	*N* = 2424	*N* = 1387	*N* = 1037	
Sex, male, n (%)	1457 (60.1)	902 (65.0)	555 (53.5)	< 0.001
Age, years, mean (SD)	60.18 (11.18)	62.00 [54.00, 68.00]	60.00 [52.00, 66.00]	< 0.001
BMI, mean (SD)	22.09 (3.13)	21.90 [19.70, 24.10]	22.30 [20.10, 24.60]	0.002
PGSGA (median [IQR])	6.83 (5.12)	7.00 [4.00, 11.00]	4.00 [2.00, 8.00]	< 0.001
KPS (median [IQR])	82.60 (13.84)	80.00 [70.00, 90.00]	90.00 [80.00, 90.00]	< 0.001
TP (median [IQR])	68.40 (7.04)	68.00 [63.05, 73.00]	69.30 [65.00, 73.40]	< 0.001
Scr (median [IQR])	69.89 (33.62)	65.40 [54.40, 79.00]	65.75 [55.90, 78.00]	0.803
Alb (median [IQR])	38.17 (5.08)	36.80 [33.35, 40.00]	40.50 [37.70, 43.00]	< 0.001
Tch (median [IQR])	4.63 (1.22)	4.36 [3.73, 5.14]	4.66 [4.01, 5.39]	< 0.001
CRP (median [IQR])	23.63 (38.74)	21.30 [9.57, 51.65]	3.02 [1.31, 3.24]	< 0.001
Glu (median [IQR])	5.86 (1.95)	5.47 [4.86, 6.40]	5.26 [4.80, 5.97]	< 0.001
Hb (median [IQR])	119.85 (29.05)	120.00 [102.00, 134.00]	130.00 [115.00, 140.00]	< 0.001
N (median [IQR])	4.82 (3.33)	4.80 [3.40, 6.66]	3.40 [2.57, 4.64]	< 0.001
L (median [IQR])	1.49 (0.82)	1.25 [0.90, 1.67]	1.59 [1.24, 1.97]	< 0.001
RBC (median [IQR])	6.06 (82.34)	4.07 [3.58, 4.53]	4.30 [3.91, 4.69]	< 0.001
PLT (median [IQR])	237.93 (98.06)	239.00 [175.50, 305.00]	213.00 [168.00, 268.00]	< 0.001
Lung cancer, yes, n (%)	917 (37.8)	539 (38.9)	378 (36.5)	< 0.001
Gastric cancer, yes, n (%)	276 (11.4)	155 (11.2)	121 (11.7)	< 0.001
Liver cancer, yes, n (%)	92 (3.8)	63 (4.5)	29 (2.8)	< 0.001
Breast cancer, yes, n (%)	136 (5.6)	53 (3.8)	83 (8.0)	< 0.001
Esophageal cancer, yes, n (%)	156 (5.95)	91 (6.5)	65 (6.2)	< 0.001
Gynecological tumor, yes, n (%)	67 (2.8)	40 (2.9)	27 (2.6)	< 0.001
Urinary cancer, yes, n (%)	62 (2.5)	40 (2.8)	22 (2.1)	< 0.001
Pancreatic cancer, yes, n (%)	97 (4.0)	57 (4.1)	40 (3.9)	< 0.001
Nasopharyngeal cancer, yes, n (%)	52 (2.1)	24 (1.7)	28 (2.7)	< 0.001
Colorectal cancer, yes, n (%)	396 (16.3)	206 (14.9)	190 (18.3)	< 0.001
Biliary Tract cancer, yes, n (%)	36 (1.5)	31 (2.2)	5 (0.5)	< 0.001
Other cancer, yes, n (%)	136 (5.6)	87 (6.3)	49 (4.7)	< 0.001
Surgery, yes, n (%)	1492 (61.6)	923 (66.5)	569 (54.9)	< 0.001
Radiotherapy, yes, n (%)	2114 (87.2)	1203 (86.7)	911 (87.8)	0.452
Chemotherapy, yes, n (%)	897 (37.0)	534 (38.5)	363 (35.0)	0.085

*BMI* Body mass index, *PG-SGA* Patients-generated subjective nutritional assessment, *KPS* Karnofsky performance status Scale, *TP* Total protein, *Scr* Serum creatinine, *Alb* Albumin, *Tch* Total cholesterol, *CRP* C-reactive protein, *Glu* Glucose, *Hb* Hemoglobin, *N* Neutrophils, *L* lymphocytes, *RBC* Red blood cell, *PLT* Platelets

**Fig. 2:**
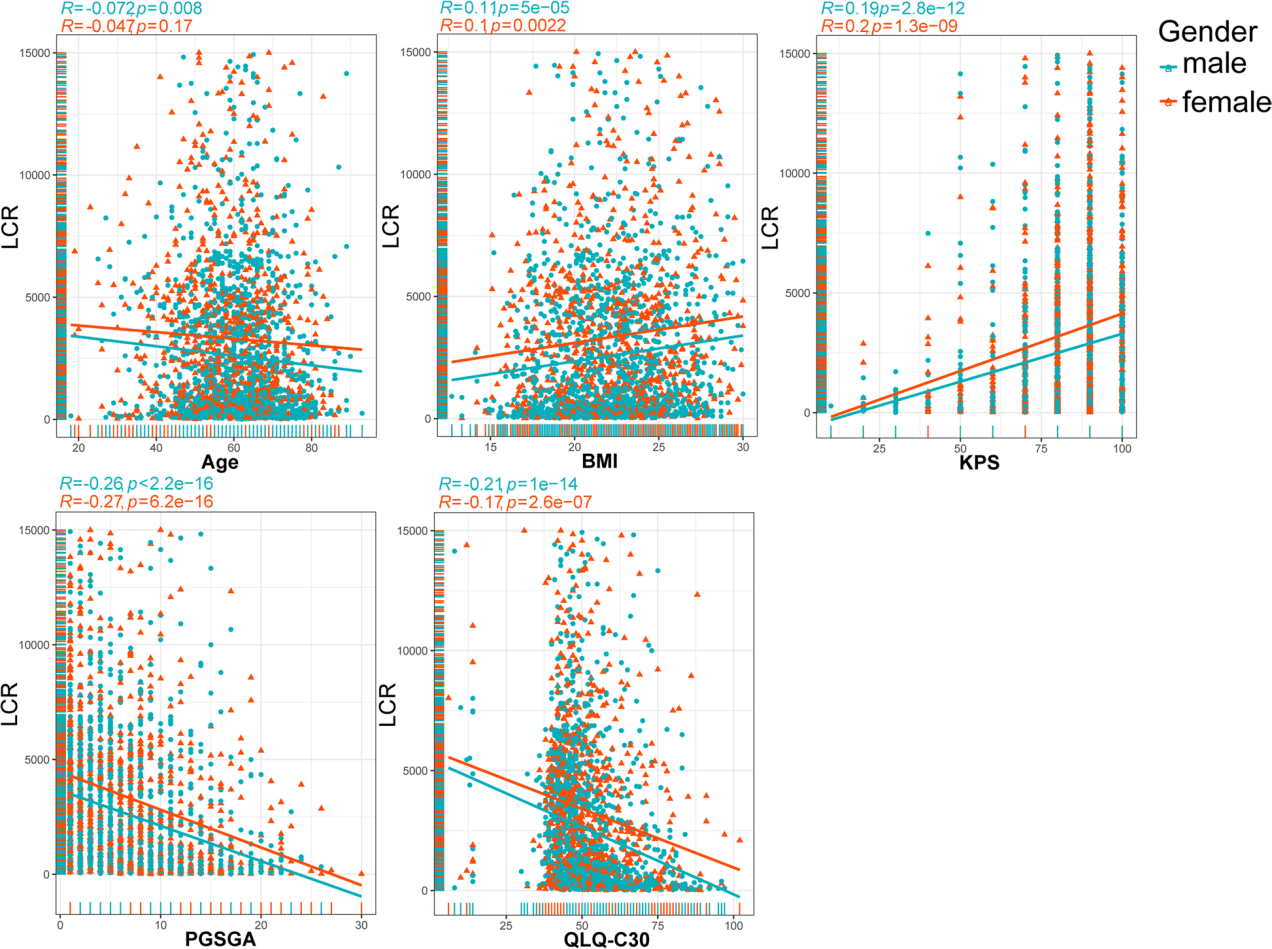
Associations between the LCR and clinical parameters. Notes: LCR, lymphocyte C-reactive protein ratio; BMI, body mass index; KPS, Karnofsky Performance Status Scale; PG-SGA, Patients-generated subjective nutritional assessment; QLQ-C30, Core Quality of Life questionnaire

**Fig. 3:**
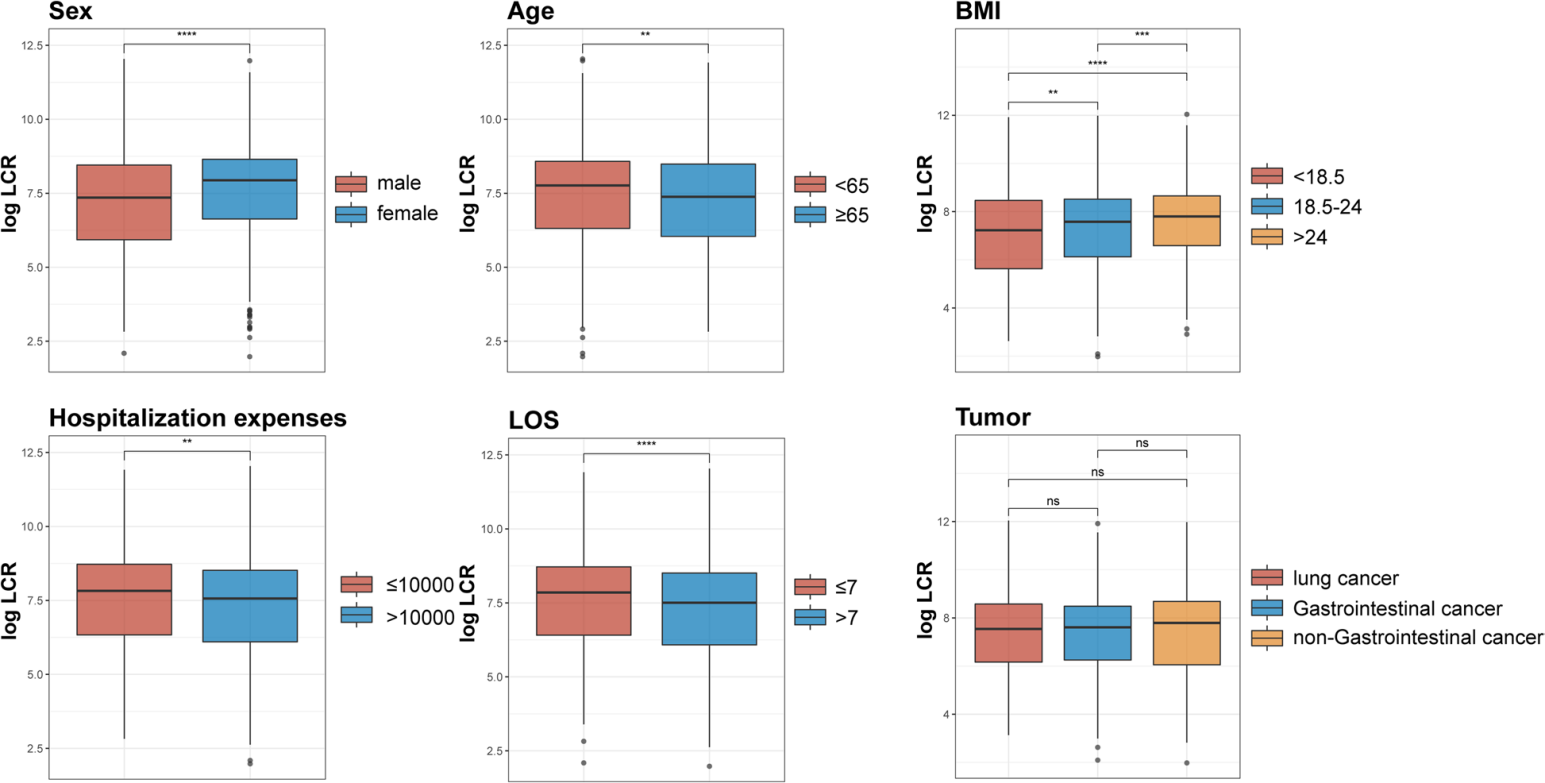
LCR in patients with TNM stage IV. Notes: LCR, Lymphocyte to C-reactive protein ratio; TNM, tumor, node, metastasis; BMI, body mass index

Furthermore, a Spearman rank correlation test was performed to compare the correlations between LCR and age, BMI, KPS, PG-SGA, and QLQ-C30 (Fig. 2). LCR was negatively correlated with age (men, *r* = -0.072; women, *r* = -0.047). The PG-SGA and QLQ-C30 exhibited a strong negative association with LCR (men: *r* = -0.26; women: *r* = -0.27; men: *r* = -0.21; women: *r* = -0.17), whereas BMI and KPS were positively associated with LCR (men: *r* = 0.11; women: *r* = 0.1; men: *r* = 0.19; women: *r* = 0.2) (Fig. 2). Furthermore, we found that LCR levels were significantly enhanced in male patients with stage IV cancer, aged < 65 years, BMI greater than 24, LOS less than 7, and hospitalization expenses less than or equal to 10,000 yuan, while there was no significant difference in LCR levels among patients with stage IV cancer with gastrointestinal tumors, non-gastrointestinal tumors, and lung cancer (Fig. 3).

### Comparison of LCR scores and current commonly used inflammation-based prognostic systems

In order to judge the prognostic value of the index from different dimensions, we used the time-dependent AUC curve to observe the time dynamic prediction ability of the index, and we also used the C index for comprehensive survival prediction. We first identified the accuracy of 15 frequently used scoring indices of patients with stage IV cancer. The LCR scores consistently had higher C-index values than the other scoring systems (See Additional file 2). The statistical differences in AUC values between LCR and other scores were also compared and showed that the AUC values of LCR were higher than those of other indicators for patients with stage IV cancer (See Additional file 3). These results demonstrated that LCR was more accurate than other inflammation-related indicators in predicting the prognosis of patients with stage IV cancer.

### Association of LCR with OS in patients with stage IV cancer

**Fig. 4:**
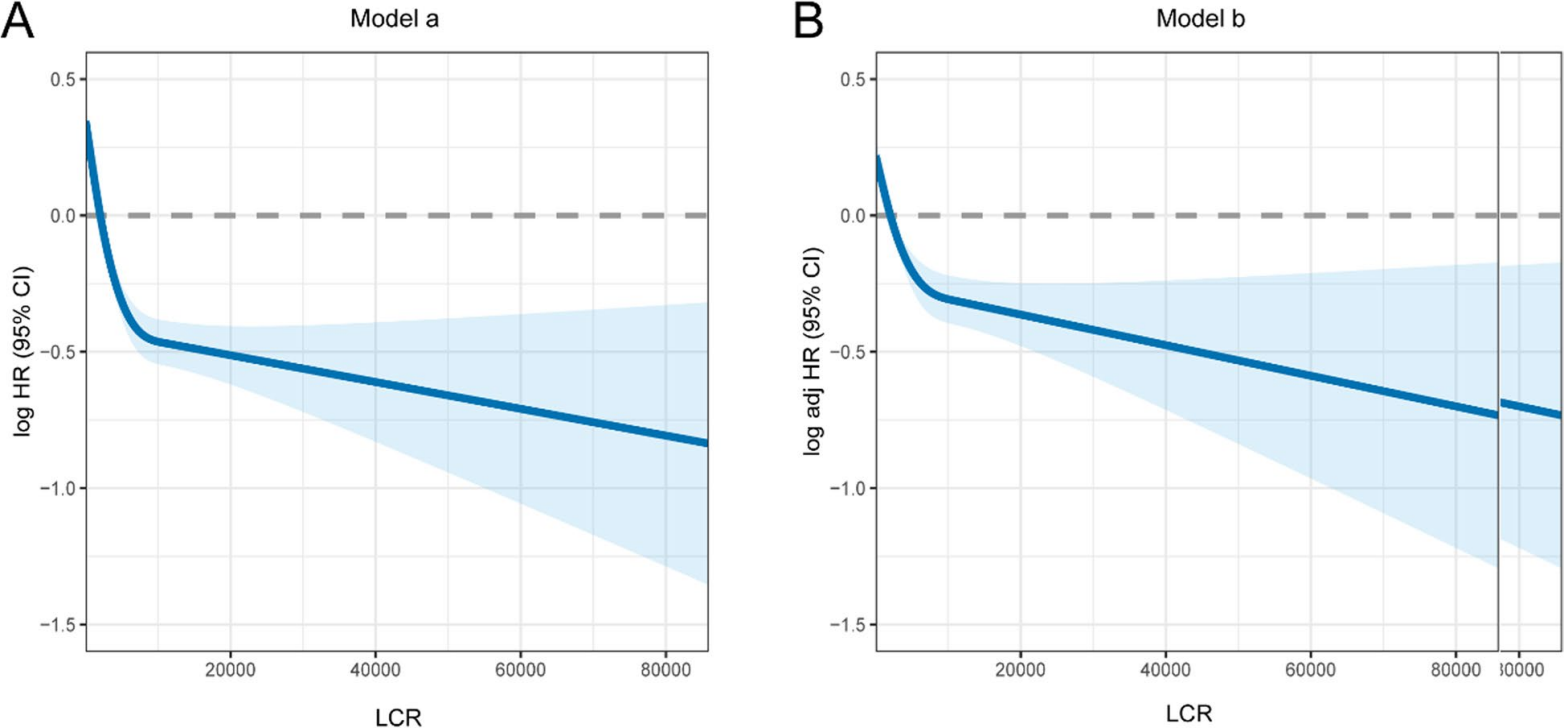
The association between LCR and hazard ratio of overall survival. Splines is adjusted by sex, age, BMI, tumor type, radiotherapy, chemotherapy, KPS score, albumin level, total bilirubin level, red blood cell count, platelet count, hand grip strength, reported reduced food intake, reported anorexia, and EORTC QLQ-C30 score. Abbreviations: LCR, lymphocyte C-reactive protein ratio; HR, hazard ratio; adj, adjusted; CI, confidence interval; BMI, body mass index; KPS, Karnofsky Performance Status Scale; QLQ-C30, EORTC Qly-C-30 questionnaire

Next, we assayed the association between LCR and the HR for OS of stage IV cancer. When analyzed as a continuous variable, the multivariate-adjusted RCS with a cubic spline function suggested that LCR had an L-shaped dose–response association with the all-cause mortality risk in patients with stage IV cancer (Fig. 4), which indicated that the lower LCR levels tended to have a worse prognosis. Furthermore, we analyzed other prognostic indicators (See Additional file 4), where NLR, PLR, GLR, SII, CAR, controlling nutritional status score, and CRP were negatively associated with prognosis, and ALI, GNRI, mGNRI, AGR, PNI, and NRI were positively associated with prognosis. In the multivariate Cox regression models (Table 2), continuous LCR as linear predictor was positively correlated with better prognosis (HR 0.84 per standard deviation increase, 95% CI: 0.77–0.91). LCR was divided into quartiles, and compared with the first quartile, the second (1.83–2.95), third (2.95–4.8) and fourth quartiles (> 4.80) were all positively correlated with a better prognosis (*p* < 0.001).

**Table 2:** Association between inflammatory burden index and overall survival of patients with patients with colorectal cancer

Variables	Model a		Model b		Model c	
	HR (95%CI)	*p* value	HR (95%CI)	*p* value	HR (95%CI)	*p* value
LCR						
Continuous (per SD)	0.824 (0.759,0.895)	< 0.001	0.837 (0.77,0.91)	< 0.001	0.848 (0.781,0.922)	< 0.001
Cutoff value		< 0.001		< 0.001		< 0.001
C1 (< 2813.953)	ref		ref		ref	
C2 (≥ 2813.953)	0.551 (0.494,0.616)		0.589 (0.526,0.659)	< 0.001	0.605 (0.54,0.678)	
Quartiles						
Q1 (< 4.08)	ref		ref		ref	
Q2 (4.08–11.37)	0.716 (0.622,0.825)	< 0.001	0.726 (0.629,0.839)	< 0.001	0.744 (0.644,0.86)	< 0.001
Q3 (11.37–65.47)	0.548 (0.473,0.635)	< 0.001	0.581 (0.5,0.675)	< 0.001	0.605 (0.52,0.704)	< 0.001
Q4 (≥ 65.47)	0.437 (0.376,0.508)	< 0.001	0.48 (0.412,0.559)	< 0.001	0.502 (0.43,0.585)	< 0.001
p for trend		< 0.001		< 0.001		< 0.001

Model a: No adjusted

Model b: Adjusted for age, sex, BMI, TNM stage

Model c: Adjusted for age, sex, BMI, TNM stage, tumor type, surgery, radiotherapy, chemotherapy, hypertension, diabetes, smoking, drinking, family history

*HR* Hazard ratio, *95%CI* 95%confidence intervals, *NLR* Neutrophil-to-lymphocyte ratio

**Fig. 5:**
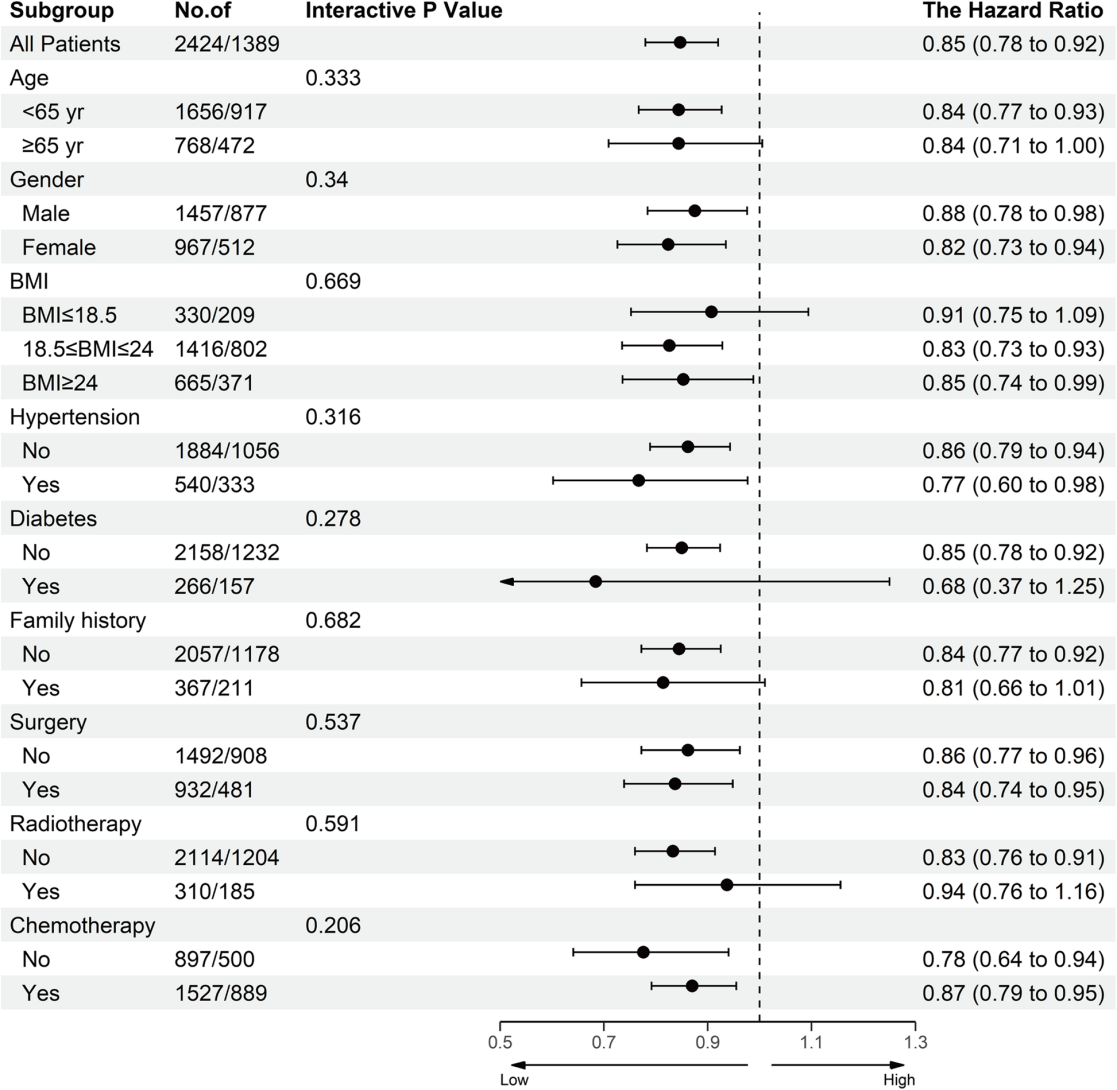
The association between LCR (stratified by cut-offs 3.3125) and hazard ratios of overall survival in various subgroups. Except the stratifying variable, the model is adjusted for sex, age, BMI, tumor type, radiotherapy, chemotherapy, KPS score, albumin level, total bilirubin level, red blood cell count, platelet count, hand grip strength, reported reduced food intake, reported anorexia, and EORTC QLQ-C30 score. Notes: LCR, lymphocyte C-reactive protein ratio; BMI, body mass index; KPS, Karnofsky Performance Status Scale; QLQ-C30, EORTC Qly-C-30 questionnaire

To further explore the potential impact of LCR on patients with stage IV cancer, we used stratified analyses to evaluate the relationship between LCR and HR of OS. As shown in Fig. 5, LCR showed a trend consistent with the main outcome in most subgroups, while there was no interaction between LCR and each subgroup factor. These results suggest that LCR can be used as an independent prognostic predictor in patients with stage IV cancer.

### LCR survival curve for patients with stage IV cancer

**Fig. 6:**
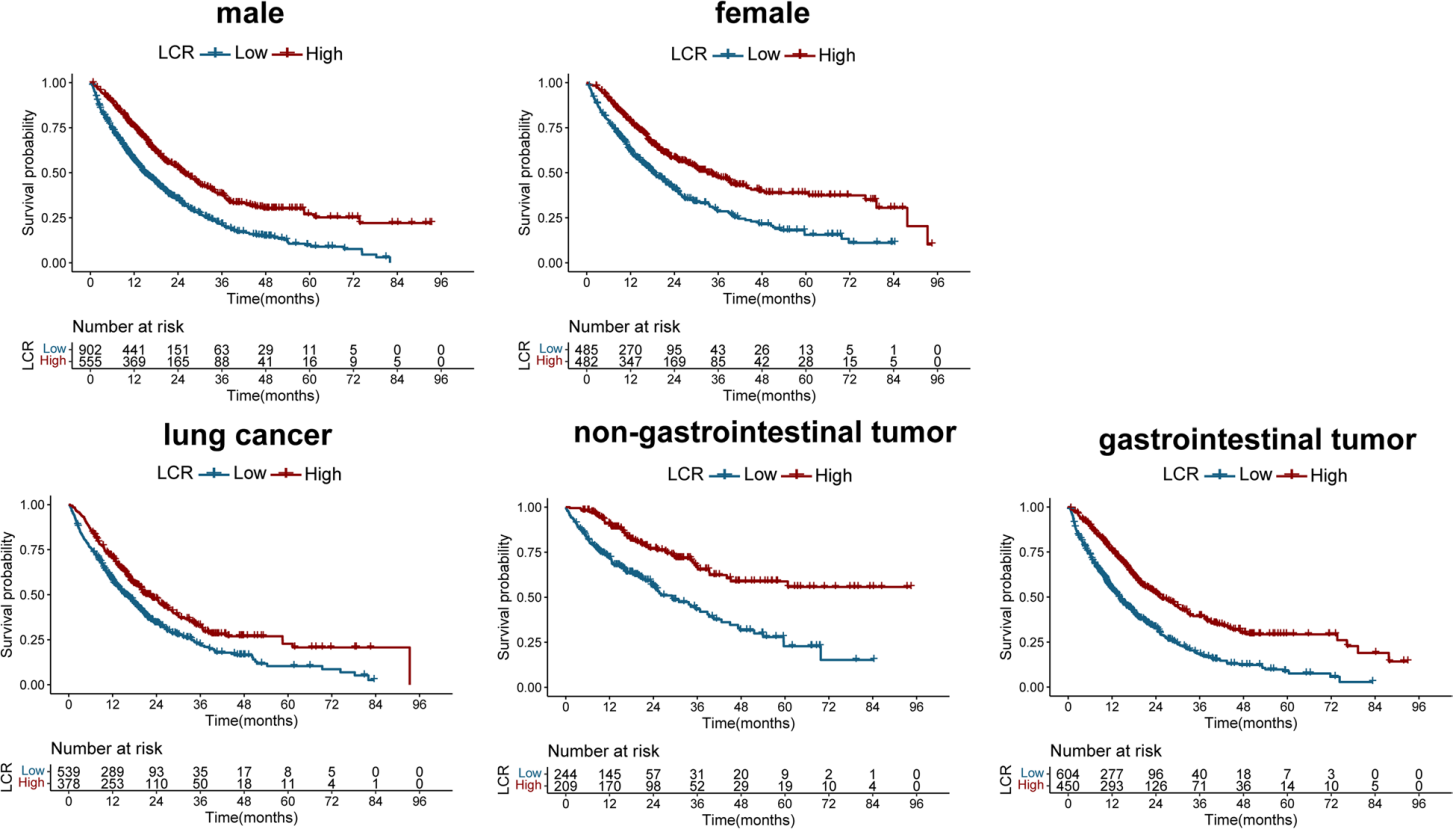
The relationship between LCR and overall survival in patients with TNM stage IV. Notes: LCR, lymphocyte C-reactive protein ratio; TNM, tumor, node, metastasis

We next used Kaplan–Meier curves and log-rank tests to evaluate time-patient survival trends and compare survival between groups. Patients with high LCR had significantly lower OS than those with low LCR (OS, 42.8% vs. 63.7%) (Fig. 6). We performed a subgroup survival analysis and found that patients with low LCR had worse survival than those with high LCR, in both men and women. Furthermore, in the subgroup analysis of different tumor types, we found that LCR could significantly stratify the prognosis of different tumor types, including gastrointestinal tumors, non-gastrointestinal tumors, and lung cancers (Fig. 6).

After 90 days of anti-cancer treatment, 199 patients died in the low LCR group, with a mortality rate of 14%, and 42 patients died in the high LCR group, with a mortality rate of 4%. Using logistic regression, we identified low LCR as an adverse factor affecting 90-day outcomes in patients with stage IV cancer (odds ratio [OR] = 1.003; 95% CI = 1.002 – 1.004, *p* < 0.001) (Table 3).

**Table 3:** Association between inflammatory burden index and overall survival of patients with patients with colorectal cancer at 90-day outcome

Variables	Model a		Model b		Model c	
	HR (95%CI)	*p* value	HR (95%CI)	*p* value	HR (95%CI)	*p* value
LCR						
Continuous (per SD)	0.525 (0.335,0.823)	0.005	0.54 (0.347,0.843)	0.007	0.56 (0.359,0.873)	0.011
Cutoff value		< 0.001		< 0.001		< 0.001
C1 (< 2813.953)	ref		ref		ref	
C2 (≥ 2813.953)	0.252 (0.179,0.355)		0.281 (0.198,0.397)	< 0.001	0.296 (0.209,0.42)	
Quartiles						
Q1 (< 4.08)	ref		ref		ref	
Q2 (4.08–11.37)	0.344 (0.244,0.485)	< 0.001	0.376 (0.265,0.533)	< 0.001	0.390 (0.274,0.553)	< 0.001
Q3 (11.37–65.47)	0.23 (0.156,0.34)	< 0.001	0.270 (0.182,0.401)	< 0.001	0.284 (0.191,0.423)	< 0.001
Q4 (≥ 65.47)	0.145 (0.092,0.229)	< 0.001	0.170 (0.107,0.270)	< 0.001	0.183 (0.115,0.293)	< 0.001
p for trend		< 0.001		< 0.001		< 0.001

Model a: No adjusted

Model b: Adjusted for age, sex, BMI, TNM stage

Model c: Adjusted for age, sex, BMI, TNM stage, tumor type, surgery, radiotherapy, chemotherapy, hypertension, diabetes, smoking, drinking, family history

*HR* Hazard ratio, *95%CI* 95%confidence intervals, *NLR* Neutrophil-to-lymphocyte ratio

## Discussion

There is increasing evidence that inflammation/nutrition-based markers are reliable predictors of OS in patients with cancer; however, the best predictors for patients with stage IV cancer are unclear. In this study, we used a large cohort to evaluate and compare 15 kinds of inflammation/nutrition-based indicators and found that LCR was more accurate in predicting the prognosis of patients with stage IV cancer than other inflammation-related indicators. In addition, our findings indicated that a low LCR was also an adverse factor affecting the 90-day prognosis of patients with stage IV cancer.

As a systemic inflammatory mediator, CRP is an evolutionarily conserved innate immune polymer protein, which has been considered as an important biomarker to predict cancer patient survival [19, 20]. CRP is mainly produced in the liver in response to cytokines released by phagocytes under the conditions of trauma, infection, inflammation, and advanced cancer [21, 22]. In patients with advanced tumors, some inflammatory factors such as tumor necrosis factor alfa, interleukin 6, and interleukin 1 beta can induce the synthesis of CRP in hepatocytes while promoting angiogenesis to support tumor growth and anti-apoptosis capabilities to protect tumor cells. Therefore, high-level CRP is positively correlated with prognosis [23, 24]. Neutral granulocyte, lymphocyte, and platelet levels reflect the severity of inflammation, and an increasing amount of evidence shows that combinations of these systemic inflammatory parameters, such as the NLR and PLR, can provide prognostic information for some malignant tumors prognostic information [25]. LCR is directly related to lymphocyte counts and CRP levels. Our results showed that low-level LCRs were negatively correlated with patients with advanced tumor.

The advantage of LCR is that it can be measured quickly, inexpensively, and non-invasively; thus, it is widely used in clinical settings. A prospective cohort study showed that LCR had the highest prognostic value prediction among all inflammation-based scores in patients with non-metastatic colorectal cancer [26]. Iseda et al. found that a high preoperative LCR correlated with a high serum albumin concentration, small tumor size, early Barcelona Clinic Liver Cancer stage, and low rates of microscopic vascular invasion and microscopic intrahepatic metastasis [27]. Other studies have shown that patients with hepatocellular carcinoma with low-LCR status had significantly worse outcomes of overall survival and disease-free survival than patients with high-LCR status [28]. These studies are consistent with our results, all of which indicate that LCR can be a useful biomarker to predict the prognosis of various cancers. However, our study included a larger sample size, evaluated a variety of cancer and inflammation indicators, and explored the relationship between LCR and the prognosis of patients with TNM stage IV for the first time. In addition, we confirmed for the first time that after 90 days of hospitalization, for patients with stage IV cancer, the number of deaths in the low LCR group was significantly higher than in the high LCR group; that is, low LCR was also an unfavorable factor affecting the 90-day outcome of patients with stage IV cancer. Therefore, LCR can be used as an accurate biomarker to predict the prognosis of patients with stage IV tumors, which allows the early stratification of patients with stage IV cancer to optimize treatment.

Compared to previous studies, our study was a multicenter prospective study with a large number of and explored the overall population of patients with stage IV cancer for the first time. However, this study had several limitations. First, there may have been selection bias in this study because of its retrospective design. Second, universality needs to be verified, because the selected study participants were all Chinese. Third, because LCR may change during treatment, it is still unclear whether this change can more accurately predict tumor results. In summary, this study may help doctors better evaluate the prognosis of patients with stage IV tumors and determine tumor follow-up strategies for patients with colorectal cancer.

## Conclusions

In conclusion, this study demonstrated that LCR was more accurate than other inflammation-related indicators in predicting the prognosis of patients with stage IV cancer. Patients with stage IV cancer with high LCR levels exhibited a better prognosis, whereas patients with low LCR levels had worse survival times. Therefore, the LCR score can be helpful for clinicians to classify patients according to their immune, inflammatory, and nutritional status; evaluate their prognosis; and make treatment and follow-up plans accordingly. Our research results may eventually provide useful information for medical guidelines.

## Supplementary Information

**Additional file 1.**

## Data Availability

All data needed to evaluate the conclusions of the study are presented in this paper and/or the Supplementary Materials. Additional data related to this study is available upon request to authors/corresponding author.
