# Supplementary material for: Lymphocyte to C-reactive protein ratio could better predict the prognosis of patients with stage IV cancer
Source: BMC Cancer. 2022 Oct 20;22:1080. doi: 10.1186/s12885-022-10145-x (PMC9585763; doi:10.1186/s12885-022-10145-x)
Supplement: Supplementary file 1 — Additional file 1. [file 12885_2022_10145_MOESM1_ESM.docx]

**Supplementary Materials**

**Additional file 1. Cut-off value of Inflammatory nutritional parameters and anthropometric indicators.**

|  |  | cut point | statistic |
| --- | --- | --- | --- |
| NLR |  | 3.311 | 9.012 |
| PLR |  | 327.184 | 4.988 |
| GLR |  | 4.350 | 5.186 |
| SII |  | 691.014 | 7.833 |
| ALI |  | 33.349 | 9.591 |
| CAR |  | 0.091 | 10.301 |
| GNRI |  | 93.342 | 8.859 |
| mGNRI |  | 42.917 | 10.116 |
| AGR |  | 1.245 | 8.209 |
| PNI |  | 39.570 | 7.798 |
| NRI |  | 94.845 | 8.837 |
| LCR |  | 2813.953 | 10.810 |
| CRP |  | 3.580 | 9.970 |

Notes: NLR, neutrophil-to-lymphocyte ratio; PLR, platelet-to-lymphocyte ratio; GLR, glucose-to-lymphocyte ratio; ALI, advanced lung cancer inflammation index; CAR, SII, systemic immune-inflammation index; CONUT, controlling nutritional status score; mGPS, modified Glasgow Prognostic Score; GNRI, Geriatric Nutritional Risk Index; AGR, albumin-globulin ratio; PNI, prognostic nutritional index; NRI, nutritional risk index; LCS, lymphocyte C-reactive protein score; LCR, lymphocyte-to-C-reactive protein (CRP) ratio

**Additional file 2. C-index of 15 Inflammatory nutritional parameters and 5 anthropometric indicators.**

|  | c-index | lower .95 | upper .95 |
| --- | --- | --- | --- |
| LCR | 0.602 | 0.586 | 0.619 |
| ALI | 0.601 | 0.585 | 0.618 |
| CAR | 0.600 | 0.583 | 0.616 |
| CRP | 0.595 | 0.579 | 0.612 |
| mGNRI | 0.592 | 0.576 | 0.609 |
| NLR | 0.588 | 0.572 | 0.605 |
| PNI | 0.586 | 0.569 | 0.603 |
| NRI | 0.585 | 0.568 | 0.602 |
| GNRI | 0.585 | 0.568 | 0.602 |
| mGPS | 0.580 | 0.565 | 0.595 |
| AGR | 0.576 | 0.560 | 0.592 |
| SII | 0.574 | 0.558 | 0.591 |
| CONUT | 0.572 | 0.556 | 0.589 |
| LCS | 0.557 | 0.543 | 0.572 |
| GLR | 0.552 | 0.535 | 0.569 |
| PLR | 0.542 | 0.525 | 0.560 |

Notes: NLR, neutrophil-to-lymphocyte ratio; PLR, platelet-to-lymphocyte ratio; GLR, glucose-to-lymphocyte ratio; ALI, advanced lung cancer inflammation index; CAR, SII, systemic immune-inflammation index; CONUT, controlling nutritional status score; mGPS, modified Glasgow Prognostic Score; GNRI, Geriatric Nutritional Risk Index; AGR, albumin-globulin ratio; PNI, prognostic nutritional index; NRI, nutritional risk index; LCS, lymphocyte C-reactive protein score; LCR, lymphocyte-to-C-reactive protein (CRP) ratio

**Additional file 3**


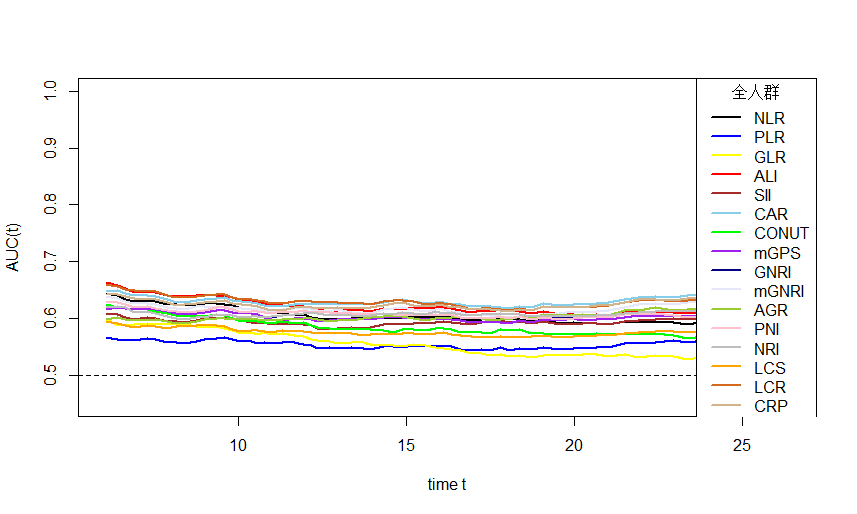


**Additional file 3.** The time-dependent AUC of systemic inflammation index and nutritional index.

Notes: NLR, neutrophil-to-lymphocyte ratio; PLR, platelet-to-lymphocyte ratio; GLR, glucose-to-lymphocyte ratio; ALI, advanced lung cancer inflammation index; CAR, SII, systemic immune-inflammation index; CONUT, controlling nutritional status score; mGPS, modified Glasgow Prognostic Score; GNRI, Geriatric Nutritional Risk Index; AGR, albumin-globulin ratio; PNI, prognostic nutritional index; NRI, nutritional risk index; LCS, lymphocyte C-reactive protein score; LCR, lymphocyte-to-C-reactive protein (CRP) ratio

**Additional file 4.**


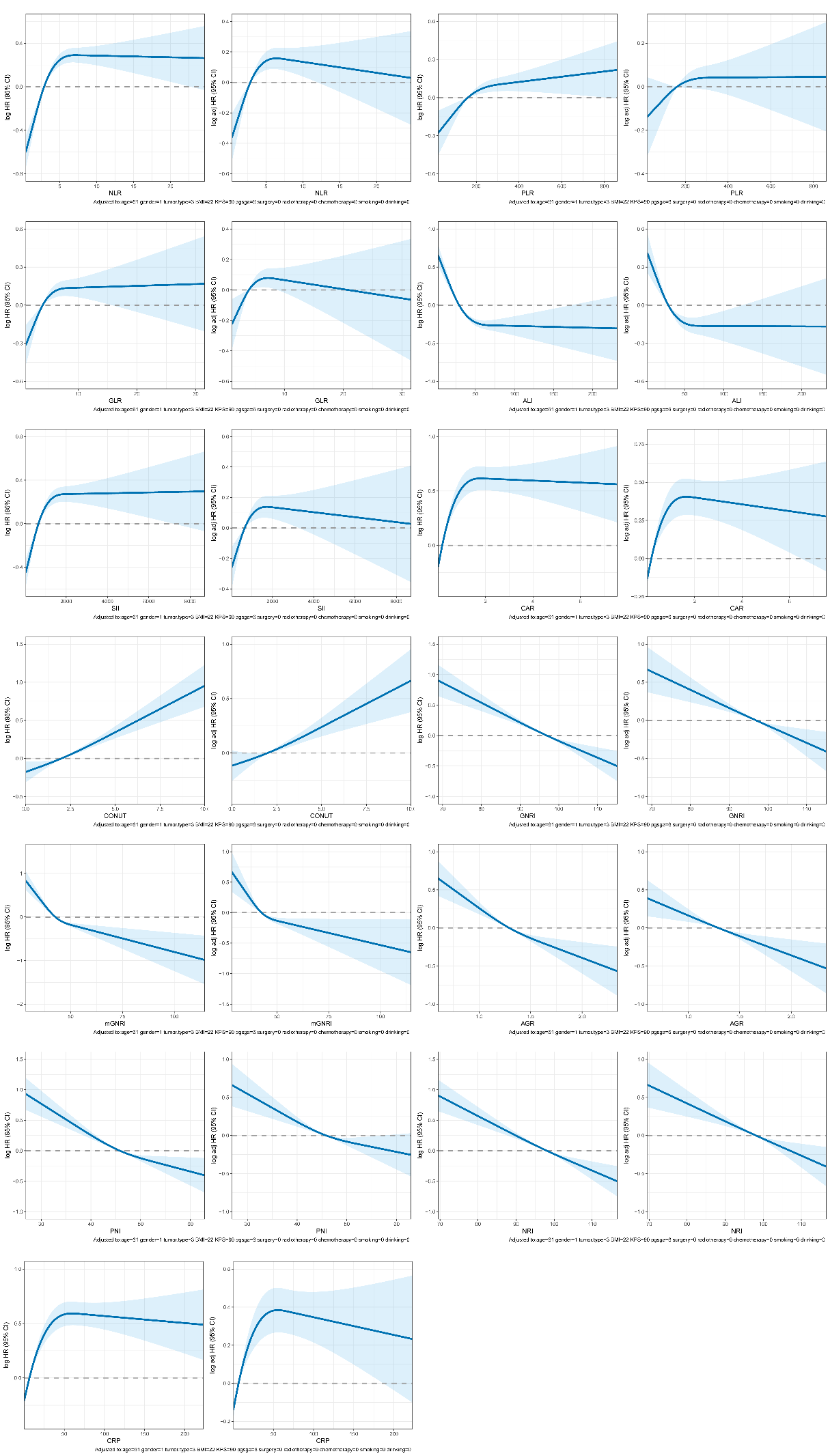


**Additional file 4.** The association between other indicators and hazard ratio of overall survival. Splines is adjusted by sex, age, BMI, tumor type, radiotherapy, chemotherapy, KPS score, albumin level, total bilirubin level, red blood cell count, platelet count, hand grip strength, reported reduced food intake, reported anorexia and EORTC QLQ-C30 score.

Notes: NLR, neutrophil-to-lymphocyte ratio; PLR, platelet-to-lymphocyte ratio; GLR, glucose-to-lymphocyte ratio; ALI, advanced lung cancer inflammation index; CAR, SII, systemic immune-inflammation index; CONUT, controlling nutritional status score; mGPS, modified Glasgow Prognostic Score; GNRI, Geriatric Nutritional Risk Index; AGR, albumin-globulin ratio; PNI, prognostic nutritional index; NRI, nutritional risk index; LCS, lymphocyte C-reactive protein score; LCR, lymphocyte-to-C-reactive protein (CRP) ratio
